# Infection susceptibility and vector competence of *Rhodnius robustus* Larrousse, 1927 and *R. pictipes* Stal, 1872 (Hemiptera, Reduviidae, Triatominae) for strains of *Trypanosoma cruzi* (Chagas, 1909) (Kinetoplastida, Trypanosomatidae) I, II and IV

**DOI:** 10.1186/s13071-022-05350-3

**Published:** 2022-06-30

**Authors:** Ana Paula de Abreu, Hevillyn Fernanda Lucas da Silva, Marcella Paula Mansano Sarto, Giullia Ferreira Iunklaus, João Vitor Trovo, Nilma de Souza Fernandes, Ana Paula Margioto Teston, Max Jean de Ornelas Toledo

**Affiliations:** 1 Programa de Pós-Graduação Em Ciências da Saúde, Centro de Ciências da Saúde (CCS), Universidade Estadual de Maringá (UEM), Maringá, PR 87020-900 Brazil; 2 Programa de Pós-Graduação Em Ciências Biológicas, Centro de Ciências Biológicas, UEM, Maringá, PR 87020-900 Brazil; 3 Departamento de Farmácia, Centro Universitario Uningá, Rodovia PR-317, Maringá, PR 87035-510 Brazil; 4 Departamento de Ciências Básicas da Saúde, CCS, UEM, Maringá, PR 87020-900 Brazil

**Keywords:** Chagas disease, DTUs, Experimental infection, Triatomines

## Abstract

**Background:**

*Rhodnius robustus* and *Rhodnius pictipes* are vectors of *Trypanosoma cruzi,* the etiologic agent of Chagas disease (CD), that are found in the Brazilian Amazon region. Susceptibility to infection and vector competence depend on the parasite-vector relationship. Our objective was to evaluate the interaction between *T. cruzi *and these two triatomine vectors in pure and mixed experimental infections of *T. cruzi* strains from the same or different geographic regions.

**Methods:**

Fifth-instar nymphs of *R. robustus* and *R. pictipes* were fed on mice infected with four *T. cruzi* strains, namely genotypes TcIAM, TcIMG, TcIIPR, and TcIVAM, respectively, from the Brazilian states of Amazonas, Minas Gerais and Paraná. Over a period of 120 days, excreta were examined every 20 days to assess vector competence, and intestinal contents (IC) were examined every 30 days to determine susceptibility to infection.

**Results:**

The highest positive rate in the fresh examination (%+FE, 30.0%), the highest number of parasitic forms (PF,* n* = 1969) and the highest metacyclogenesis rate (%MC, 53.8%) in the excreta were recorded for *R. robustus*/TcIVAM. Examination of the IC of *R. pictipes* revealed a higher number of PF in infections with TcIAM (22,680 PF) and TcIIPR (19,845 PF) alone or in association (17,145 PF), as well as a %+FE of 75.0% with TcII, in comparison with the other genotypes. The highest %MC (100%) was recorded for the mixed infections of TcIAM with TcIIPR or TcIVAM in the IC of *R. pictipes*.

**Conclusions:**

Overall, both species were found to be susceptible to the *T. cruzi* strains studied. *Rhodnius robustus* showed vector competence for genotypes TcIVAM and TcIAM+TcIVAM and *R. pictipes* for TcIAM+TcIVAM and TcIAM+TcIIPR; there was elimination of infective forms as early as at 20 days. Our results suggest that both the genetics of the parasite and its geographic origin influence the susceptibility to infection and vector competence, alone or in association.

**Graphical Abstract:**

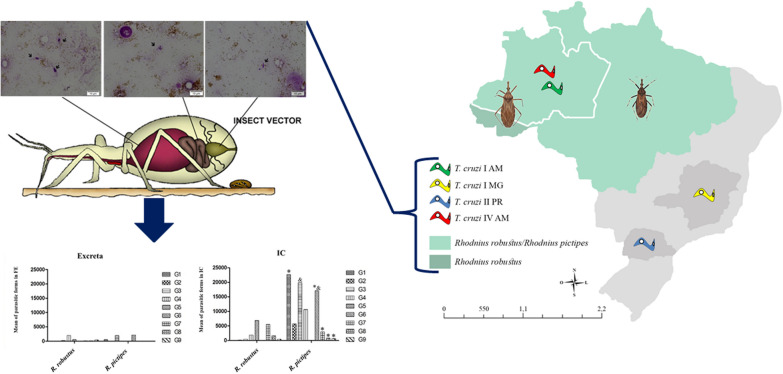

## Background

*Trypanosoma cruzi* is a hemoflagellate protozoan that is the etiologic agent of Chagas disease (CD), also known as American trypanosomiasis [1]. Triatomine insects (Hemiptera, Reduviidae, Triatominae) serve as vectors, while the vertebrate hosts can be wild and domestic mammals, as well as humans [2]. While CD is endemic in Latin America, globalization and human migrations have been major factors in its spread to countries considered to be non-endemic where it has become an important disease. According to the WHO, 6–7 million people are infected with CD worldwide [3], and it is estimated that 62.4% of these reside in the countries belonging to the Southern Cone of South America (Argentina, Brazil, Chile, Paraguay and Uruguay) [4].

This parasite has a wide genetic diversity, and its strains are classified into six discrete typing units (DTUs), TcI to TcVI [5, 6]. *Trypanosoma cruzi* I (TcI) is the DTU most frequently isolated from the wild, and its presence has been reported in different triatomine species of the genus *Rhodnius* Stål, 1859. This strain typically causes heart disease in northern South America [5]. TcII has been isolated from triatomines of the genus *Panstrongylus* Berg, 1879 and wild animals (*Didelphis* sp. and *Euphractus* sp.). This strain generally causes cardiac and digestive CD in infected persons in southern South America (South Cone countries) and occurs more frequently in the domestic transmission cycle [5]. TcIII and TcIV are less frequently isolated from the wild and are associated with outbreaks of oral CD in the Amazon, with the latter associated with heart disease in northern South America. TcIV has only been isolated from the species *Rhodnius robustus* Larrousse, 1927 and *Rhodnius brethesi* Matta, 1919. TcV and TcVI are considered to be hybrid strains that occur in the domestic transmission cycle. Recently, TcBat, the seventh DTU, was identified. This genetic lineage is associated with bats, based on its detection in both insect and fruit-eating bats in China, but its presence in humans has already been demonstrated by molecular techniques. Phylogenetic analysis, based on PCR amplification of a multicopy amplicon targeting members of the trans-sialidase family and large-scale sequencing, has confirmed that TcBat is indeed a DTU distinct from the others and suggests a scenario in which it is more closely related to TcIII than to TcI [7–10]. As yet, there are few studies on TcBat and its possible clinical manifestations in humans [11].

The vectors of CD are arthropods belonging to the Triatominae subfamily, which are distributed in five tribes and 18 genera, encompassing around 157 species (154 living species and three fossils) [12–16]. The genera *Panstrongylus* and *Triatoma* Laporte, 1832, as well as the genus *Rhodnius*, play an important role in the epidemiology of CD. The genus *Rhodnius* comprises 21 species [16–18], including *R. robustus* and *R. pictipes* Stal, 1872*,* which inhabit different species of wild and peri-urban palm trees. The winged adults can invade human dwellings and their dependencies without colonizing them [19, 20]. In the Brazilian state of Amazonas, palm tree populations act as sources of adult adventitious bugs of *R. robustus *sensu lato, *R. pictipes* and *R. brethesi* (these species have high rates of infection by trypanosomatids), leading to the contamination of food-processing equipment and, subsequently, the emergence of acute cases of oral CD, or to the attack of forest workers by these bugs, leading to isolated cases of vector transmission [19, 21–23].

In studies on the interaction of different strains of *T. cruzi* with the main vector species, variations in both protozoan population density and insect infection rates have been reported [2, 24–26]. The mean positivity rates among different triatomine species ranged from 9.8% in *Triatoma dimidiata* Latreille, 1811 to 91.4% in *Triatoma pseudomaculata* Corrêa & Espínola, 1964, with an overall mean positivity rate of 67.3% [2].

Although the complex interaction between *T. cruzi* and its vector for transmissibility is not yet fully understood, it is known that this interaction can be influenced by the biological, biochemical and genetic characteristics of both the vector and the parasite, combined with the fact there are sympatric species that are better adapted to the environment in which they are inserted [26–29]. In addition, the hematophagous habits of the vectors contribute to the selection of populations of *T. cruzi*, in that some genetic lineages can be eliminated while in others an increase in the population can occur, even when they are concomitant in the vector [30]. In this context, the objective of the present study was to evaluate the susceptibility to infection and vector competence of triatomine species from the Amazon region, both with strains from the same region and with those from other geographic areas of Brazil.

## Methods

### Ethical aspects

The use, maintenance and care of the mice in the present study followed the guidelines of the Conselho Nacional de Controle de Experimentação Animal (CONCEA), and the project was approved by the Comissão de Ética no Uso de Animais of Universidade Estadual de Maringá (CEUA/UEM) (Registration no. 9659251017). The use of *T. cruzi* strains obtained from humans was approved by the Ethics Committees of the Fundação de Medicina Tropical Doutor Heitor Vieira Dourado (Registration no. 360/07) and of UEM (Registration no. 100/04 e 375/07).

### Triatomines and their maintenance

The colonies of *R. robustus* species used in this study are derived from Loreto, Peru [31], and those of *R. pictipes* species were originally from Serra Norte, Pará State, Brazil [32]. *Rhodnius robustus* and *R. pictipes* were kindly donated by the Laboratório Nacional e Internacional de Referência em Taxonomia de Triatomíneos, at Instituto Oswaldo Cruz/Fiocruz. The insects were kept under controlled conditions of temperature (appox. 26 °C), relative humidity (approx. 60%) and light (light/dark cycle: 12/12 h), and regularly fed on Swiss mice previously anesthetized in the Parasitology Sector of the Department of Sciences Health Basics of Universidade Estadual de Maringá (UEM).

### *T. cruzi* strains

Four *T. cruzi* strains belonging to three different DTUs (TcI, TcII and TcIV) and originating from three Brazilian states (Amazonas, Minas Gerais and Paraná) were used. Two strains belong to the DTU TcI: PR150, isolated in 1990 from a patient in the chronic phase of the disease in Paraná, but originally acquired in Minas Gerais (TcIMG) [33], and AM33, isolated from *R. pictipes* captured in Manaus, Amazonas (TcIAM) in 2007 [34]. The other strains used include one belonging to DTU TcII, PR1256, which was isolated from a chronic case in Paraná in 1992 (TcIIPR) [35], and one belonging to DTU TcIV, AM14, which was isolated from an acute case acquired orally in Coari, Amazonas in 2007 (TcIVAM) [34].

### Inoculation of animals

Male Swiss *Mus musculus* mice aged 21–28 days and weighing between 18 and 22 g were used. The mice were housed in polypropylene cages maintained under a 12 h/12-h light/dark cycle; food and water were available ad libitum. The inoculum consisted 2 × 10^6^ culture-derived metacyclic trypomastigotes (CMT) from each of the four strains used in the study, in 1.0 ml of liver infusion tryptose (LIT) culture medium [36]. The mice were infected through intraperitoneal injection of the inoculum. In mixed infections, two strains were used in each injection, with equal amounts of each strain.

### Infection of triatomines

Triatomine species from the Amazon region (*R. robustus* and *R. pictipes*) were infected with strains of *T. cruzi* from the same region and from other geographic areas (Minas Gerais and Paraná States). In total, 180 fifth-instar nymphs of each species were used. As a negative control in the PCR analysis, 20 uninfected nymphs of each species were used.

**Fig. 1:**
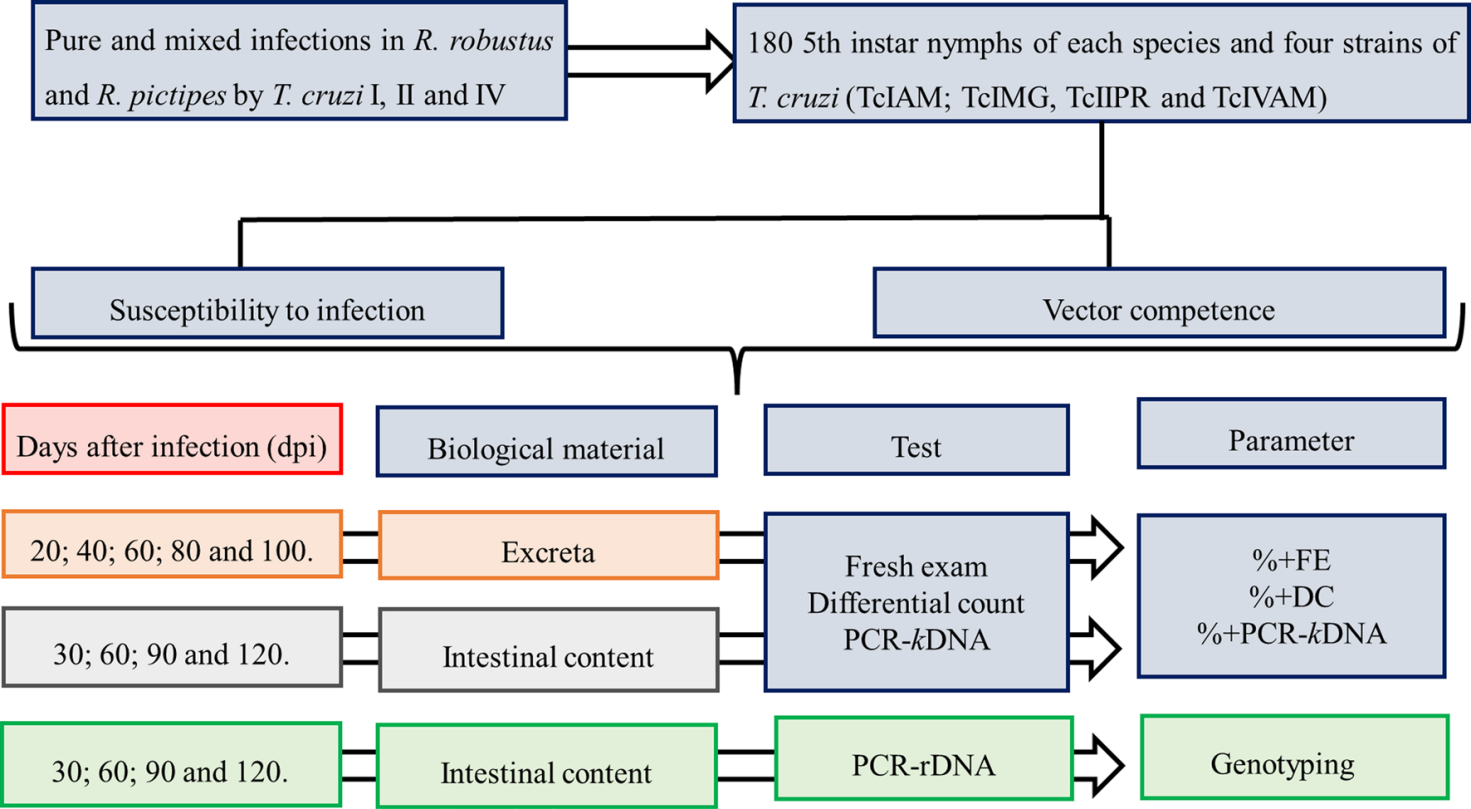
Flowchart of the experimental design. The first line provides information on the composition of the *Trypanosoma cruzi* infection (pure and mixed) of fifth-instar nymphs of two species of *Rhodnius*. Following infection, susceptibility to infection and vector competence were evaluated through tests, which provided different parameters of the different biological materials (blue boxes) on different dpi (red box). Abbreviations: DC, Differential count; dpi, days post-infection; FE, fresh examination;* k*DNA, kinetoplast DNA; rDNA, ribosomal DNA

For each triatomine species, the fifth-instar nymphs were divided into groups of 20 specimens each, for a total of nine experimental groups: (i) four groups fed on mice inoculated with a single strain of *T. cruzi* (pure infections); (ii) five groups fed on mice inoculated with two strains at the same time (mixed infections); (iii) and one group was uninfected (control group: fed on healthy mice). The groups (G) were arranged as follows: G1 (TcIAM), G2 (TcIMG), G3 (TcIIPR), G4 (TcIVAM), G5 (TcIAM+TcIVAM), G6 (TcIAM+TcIIPR), G7 (TcIMG+TcIIPR), G8 (TcIMG+TcIVAM) and G9 (TcIIPR + TcIVAM), for both triatomine species (Fig. 1).

Excreta were examined every 20 days for up to 100 days to assess vector competence, and intestinal content (IC) was examined every 30 days for 120 days to determine susceptibility to infection.

Insects from groups G1 to G9 were fed on mice previously inoculated via the intraperitoneal route, on the day of maximum parasitemia peak. In pure infections, the maximum peak of parasitemia occurs on the seventh day post-infection (dpi) for strains AM33 (TcIAM) and AM14 (TcIVAM), and on the 13th dpi for strains PR150 (TcIMG) and PR1256 (TcIIPR). In mixed infections (TcIAM+TcIIPR, TcIAM+TcIVAM, TcIMG+TcIIPR, TcIMG+TcIVAM and TcIIPR+TcIVAM), the insects were fed both on the seventh and 13th dpi, according to the parasitemia curve profiles of each strain, which our group has previously described [33, 37, 38]. At the peak of parasitemia, the mice had approximately 1400 blood trypomastigotes (BT) per 0.1 ml of blood [37, 39], both for the pure infections with one strain (TcIAM, TcIMG, TcIIPR or TcIVAM) and for the mixed infections containing two strains (TcIAM+TcIIPR, TcIAM+TcIVAM, TcIMG+TcIIPR, TcIMG+TcIVAM and TcII+TcIVAM).

The triatomines were kept under controlled conditions of temperature (28 ± 1 °C), relative humidity (60 ± 5%) and photoregimen (12/12-h light/dark), in a Biochemical Oxygen Demand (BOD) in the Parasitology Sector of the Department of Basic Health Sciences.

### Determination of blood volume and number of forms ingested

The nymphs were weighed individually in a group of 20 insects for each experimental group, with a semi-analytical scale (model BK 300; Gehaka, São Paulo, Brazil) before and after the blood meal. Nymphs with a weight gain ≥ 1.0 mg after feeding were considered to be fed. To estimate both the blood volume and the number of ingested parasites, a 1.0 mg weight gain was considered to be equivalent to 1.0 µl of ingested blood [25].

The mean number of blood forms ingested was determined from the mean blood volume ingested per group × 1400 BT/0.1 ml of blood [25].

### Analysis of excreta to determine vector competence

Triatomines were fed every 20 days with blood from uninfected mice sedated with a solution of ketamine (100 mg/kg) + xylazine (5–10 mg/kg) (1:2), according to CONCEA guidelines, and monitored for a period of 120 days. During or after the blood meal, the excreta (feces and urine) eliminated spontaneously were collected and added to 100 μl of phosphate-buffered saline (PBS) at 0.15 M (pH 7.2). A 5-μl aliquot from the pool of excreta was used to perform the fresh examination (FE), in duplicate, under an optical microscope, at a magnification of 400× [39].

A 10-μl aliquot of the pool of excreta was used to prepare smears stained with Giemsa for the differential count (DC), in duplicate, to determine the number of metacyclic trypomastigote (MT), epimastigote (EP) and spheromatigote (SF) forms per group, reading the entire smear with an immersion objective (magnification: 1000×) [40, 41]. The metacyclogenesis rate (%MT; the proportion of metacyclic forms in relation to the total number of parasitic forms) was computed considering only the EP and MT forms and calculated for each experimental group [40, 41].

### Analysis of IC to determine susceptibility to infection

Every 30 days, for a period of 120 days, the intestines of five specimens from each group were removed and macerated with 100 μl of PBS at 0.15 M, following which a 5-μl aliquot of the macerate was freshly examined (FE) [39] and a 10-μl aliquot were used to make a smear stained with Giemsa, in duplicate. The parameters to be observed were the same as described above for excreta. The samples were added to 500 µl of 70% alcohol, which were stored at −20  ºC until DNA extraction.

### PCR analysis of *T. cruzi* kinetoplast DNA

Kinetoplast DNA–PCR (*k*DNA-PCR) was used to verify the infection of negative insects in the FE. For this, the DNA of *T. cruzi* was extracted from the pool of excreta and IC of the insects using the conventional method of phenol/chloroform described by Macedo et al. [42]. Primers 121 (5′-AAATAATGTACGGG[T/G]GAGATGCATGA-3′) and 122 (5′-GGTTCGATTGGGGTTGGTGTAATATA-3′) were used to amplify (35 cycles; Techne™ TC-512 thermocycler; Thermo Fisher Scientific, Waltham, MA, USA) a 330-bp fragment of the *T. cruzi k*DNA). PCR products were visualized on a 4.5% polyacrylamide gel by silver staining, as described by Miyamoto et al. [43]. The 100-bp DNA molecular weight marker from Invitrogen™ (Thermo Fisher Scientific) was used to confirm the correct size of the analyzed fragment.

### Restriction fragment length polymorphism of the cytochrome oxidase subunit II gene

Amplification of the cytochrome oxidase subunit II (COXII) gene by PCR followed by restriction fragment length polymorphism (RFLP) analysis with the restriction enzyme AluI was used to differentiate DTUs I to VI. The amplification of the COXII gene was performed according to Abolis et al. [35] and Sá et al. [44], using primers Tcmit-10 (5′-CCATATATTGTTGCATTATT-3′) and Tcmit-21 (5′-TTGTAATAGGAGTCATGTTT-3′). The amplified product was digested with AluI and the fragments visualized in a 6% polyacrylamide gel by silver staining [44].

### 24Sα ribosomal DNA gene PCR

The PCR analysis of the 24Sα rDNA gene (rDNA-PCR) described by Souto et al. [45] was used to discriminate the *T. cruzi* DTUs present in the IC from the groups of insects infected with two strains. The size of the amplified products of this gene for the *T. cruzi* strains belonging to the six DTUs is 110 bp (TcI, TcIII and TcV), 120 bp (TcIV) and 125 bp (TcII and TcVI), respectively [6, 45, 46]. This marker enables the three DTUs of the study present in the IC of the nymphs with mixed infection to be discriminated and identified. Amplification was performed with primers D71 (5′-AAGGTGCGTCGACAGTGTGG–3′) and D72 (5′-TTTTCAGAATGGCCGAACAGT–3′), and the amplicons were run in a 6% polyacrylamide gel and visualized by silver staining [44].

### Determination of infectiousness and mortality rates

The pool of insects that presented a positive result in at least one of the methods applied (FE, DC and *k*DNA-PCR) in the excreta or IC was considered to be infected.

Deaths were recorded throughout the study period to determine the cumulative mortality rate in each experimental group.

### Statistical analysis

Data were tabulated in a Microsoft Excel 2019 spreadsheet (Microsoft Corp., Redmond, WA, USA) and statistically analyzed using BioEstat 5.0 and GraphPad Prism 8.3.1 (GraphPad Software Incs., San Diego, CA, USA) softwares. Comparisons of means and standard deviations (SD) were performed for quantitative variables. Statistical comparisons were performed between the two triatomine species (*R. robustus* × *R. pictipes*) and between groups of pure and mixed infections containing the same strains (G1×G5, G1×G6, G2×G7, G2×G8, G3×G6, G3×G9, G4×G5, G4×G7, G4×G9), comparing groups two by two or all groups at the same time. Comparisons were also made between pure and mixed infections for each species and between species, both in the excreta and in the IC. The Mann-Whitney, Wilcoxon, Student's t and Shapiro–Wilk nonparametric tests were used to compare groups. For qualitative variables, analysis of variance and Bonferroni tests were used. The significance level adopted was 5%, with associations of *P* ≤ 0.05 considered to be significant.

## Results

### Blood volume and mean number of blood trypomastigotes ingested

The pool of fifth-instar *R. robustus* and *R. pictipes* nymphs in all groups (G1–G9) showed a significant body weight gain (*P* = 0.007) following the blood meal (Table 1) with the nymphs of *R. robustus* having a significantly higher mean weight (*P* < 0.007) than those of *R. pictipes* (Table 1).

**Table 1:** Mean body weight gain after the infective meal and the mean number of blood trypomastigotes ingested by the fifth-instar nymph pool of *Rhodnius robustus* and *Rhodnius pictipes* fed on Swiss mice previously infected with 1400 BT of pure inoculum or mixed inoculum

Experimental group^a^	*Rhodnius robustus*		*Rhodnius pictipes*	
	Mean weight gain ± SD (mg)	Mean number of BT ingested	Mean weight gain ± SD (mg)	Mean number of BT ingested
G1 (TcIAM)	1096 ± 693	76,720	476 ± 399	33,320
G2 (TcIMG)	992 ± 219	69,440	628 ± 272	43,960
G3 (TcII)	1014 ± 387	70,980	687 ± 653	48,090
G4 (TcIV)	275 ± 85	19,250	452 ± 288	31,640
G5 TcIAM+TcIV)	640 ± 1110	44,800	50 ± 10	3500
G6 (TcIAM+TcII)	388 ± 35	27,160	296 ± 86	20,720
G7 (TcIMG+TcIV)	403 ± 458	28,210	571 ± 220	39,970
G8 (TcIMG+TcII)	657 ± 295	45,990	535 ± 365	37,450
G9 (TcII+TcIV)	258 ± 400	18,060	246 ± 116	17,220
Mean ± SD	730 ± 165	44,512 ± 23,093	465 ± 36	30,652 ± 14,312
*P* value	0.007*	ns	0.007*	ns

*BT* Blood trypomastigotes,* G* experimental groups,* ns* not significant, * SD* standard deviation,* TcI, TcII, TcIV* discrete typing units

*The Wilcoxon test was used to compare differences in weight gain between experimental groups (G1–G9) after the infective meal, considering a significance level of 5%

^a^Experimental groups consisted of mice infected with pure inoculum, consisting of a single strain of *Trypanosoma cruzi* (G1–G4), or with mixed inoculum, consisting of mixed strains of *T. cruzi* (G5–G9)

The *R. robustus*/TcIAM group (G1) showed the greatest increase in body weight, ingesting a mean of 1096 mg of blood, which is equivalent to 76,720 BT/µl, when compared between the nine groups for the same vector species and between vector species for the same experimental blood meal (Table 1). Among the *R. pictipes* groups, the greatest increase in body weight was recorded for the *R. pictipes*/TcIIPR group (G3), which ingested a mean of 687 mg of blood, corresponding to 48,090 BT/µl (Table 1). Despite the significant variation in weight gain between the vector species and even between the groups of the same species, we concluded that all insects were fed.

### Fresh examination, differential count and *k*DNA-PCR positivity

*Rhodnius robustus* showed FE positivity rates (%+FE) in the excreta that ranged from 0.0% in G1 (TcIAM) and G6 (TcIAM+TcIIPR) to 30.0% in G4 (TcIVAM) (*P* = 0.0116), and in the IC from 0.0% in the same groups, G1 and G6, to 37.5% in G8 (TcIMG+TcIIPR), with no significant difference in the latter when IC was compared (Table 2). The DC positivity rates (%+DC) in the excreta ranged from 0.0% in seven of the nine groups (G1-G3, G6-G9) to 30.0% in G5 (TcIAM+TcIVAM) (*P* < 0.0001), and in the IC from 0.0% in G6 to 75.0% in G5; again, the difference was not significant for the IC samples. For *k*DNA-PCR, positivity rates varied significantly for both biological materials analyzed, demonstrating its greater sensitivity. In the excreta from *R. robustus*, the positivity rates ranged from 80.0% in G7 (TcIMG+TcIVAM) to 100.0% in six of the nine groups (G1, G3, G5, G6, G8 and G9) (*P* = 0.001), while in the IC, the positivity rates ranged from 50.0% in G2 (TcIMG) to 87.5% in four of the nine groups (G3-G6) (*P* = 0.039) (Table 2).

**Table 2:** Positivity rate by fresh examination, differential count and kinetoplast DNA–PCR analysis in excreta and intestinal contents of *R. robustus* and *R. pictipes* fed on Swiss mice previously infected with 1400 blood trypomastigotes of different *Trypanosoma cruzi* strains as pure or mixed inocula

Experimental group (*n* = 20)	*Rhodnius robustus*						*Rhodnius pictipes*					
	% of positivity of excreta^a^			% of positivity of IC^a^			% of positivity of excreta^a^			% of positivity of IC^a^		
	FE	DC	*k*DNA-PCR	FE	DC	*k*DNA-PCR	FE	DC	*k*DNA-PCR	FE	DC	*k*DNA-PCR
G1 (TcIAM)	0.0	0.0	100.0	0.0	12.5	75.0	10.0	30.0	70.0	37.5	50.0	75.0
G2 (TcIMG)	12.5	0.0	90.0	12.5	12.5	50.0	0.0	0.0	90.0	12.5	50.0	87.5
G3 (TcII)	10.0	0.0	100.0	25.0	25.0	87.5	10.0	0.0	70.0	75.0	87.5	100.0
G4 (TcIV)	30.0	20.0	90.0	25.0	37.5	87.5	0.0	10.0	70.0	37.5	75.0	75.0
G5(TcIAM+TcIV)	10.0	30.0	100.0	25.0	75.0	87.5	0.0	10.0	100.0	0.0	0.0	100.0
G6 (TcIAM+TcII)	0.0	0.0	100.0	0.0	0.0	87.5	20.0	10.0	100.0	37.5	37.5	100.0
G7 (TcIMG+TcIV)	10.0	0.0	80.0	25.0	50.0	62.5	0.0	0.0	60.0	25.0	25.0	100.0
G8 (TcIMG+TcII)	10.0	0.0	100.0	37.5	37.5	75.0	0.0	0.0	100.0	25.0	12.5	37.5
G9 (TcII+TcIV)	10.0	0.0	100.0	12.5	12.5	75.0	0.0	0.0	80.0	12.5	25.0	37.5
*P* value*	0.0116	< 0.0001	0.001	ns	ns	0.039	0.001	0.002	ns	ns	ns	0.012

*DC* Differential count,* FE* fresh examination,* kDNA-PCR* kinetoplast DNA–PCR analysis

*Shapiro–Wilk test, considering a significance level of 5%. Experiments performed in duplicate

^a^The excreta pool results were obtained from five collections (at 20, 40, 60, 80 and 100 days post-infection (dpi)), and the IC pool results were obtained from four collections (at 30, 60, 90, 120 dpi)

For *R. pictipes*, the %+FE in the excreta ranged from 0.0% in six of the nine groups (G2, G4, G5, G7-G9) to 20.0% in G6 (*P* = 0.001) and in the IC, with no significant difference, from 0.0% in G5 to 75.0% in G3 (TcIIPR) (Table 2). The %+DC varied significantly in the excreta (*P*  = 0.002), from 0.0% in five of the nine groups (G2, G3, G7–G9) to 30.0% in G1, while *k*DNA-PCR positivity varied significantly in the IC (*P* = 0.012), from 37.5% in G8 and G9 to 100.0% in four groups (G3, G5–G7) (Table 2).

### Comparisons of the mean number of parasitic forms in the FE of excreta and IC

**Fig. 2:**
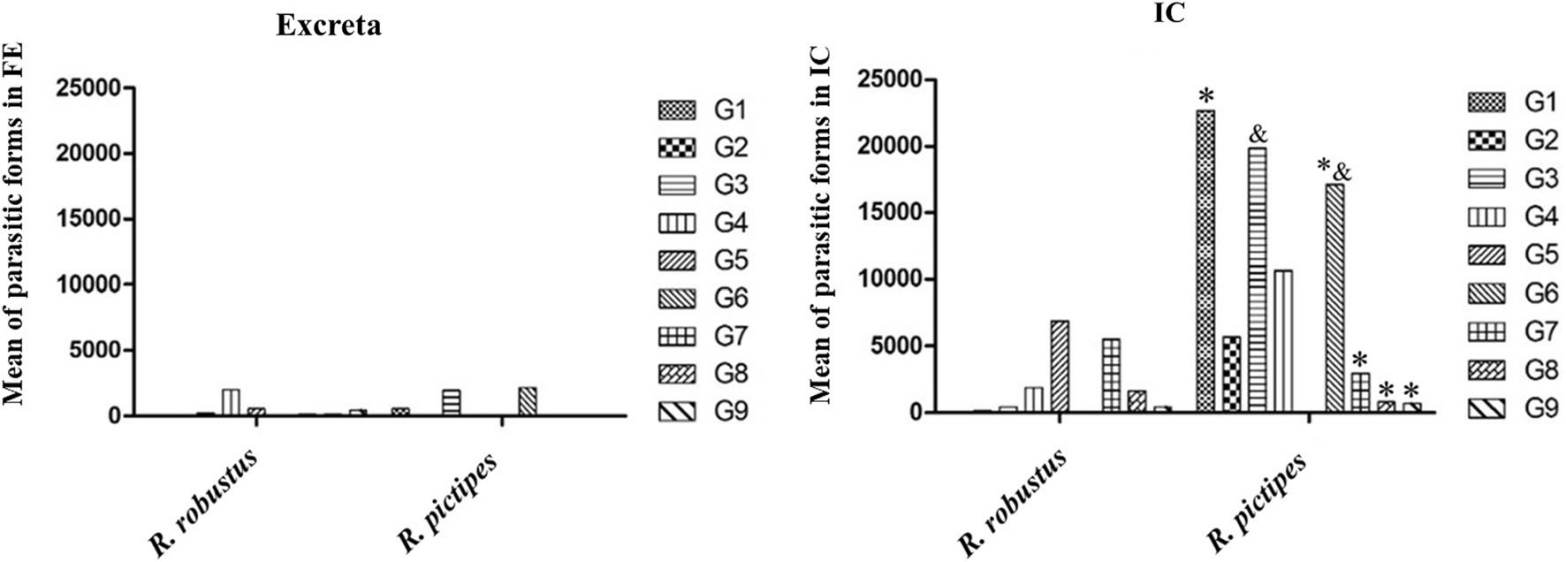
Mean number of parasitic forms on fresh examination of excreta and intestinal contents of fifth-instar *Rhodnius robustus* and *R. pictipes* nymphs infected with blood-fed *Trypanosoma cruzi* trypomastigotes in pure [G1 (TcIAM), G2 (TcIMG), G3 (TcIIPR) and G4 (TcIVAM)] and mixed infections [G5 (TcIAM+TcIVAM), G6 (TcIAM+TcIIPR), G7 (TcIMG+TcIVAM), G8 (TcIMG+TcIIPR) and G9 (TcIIPR + TcIVAM)]. Bars with the same symbols (* or &) are statistically different from each other at* P* < 0.05. Abbreviations: IC, Intestinal contents

The mean number of parasitic forms (PF) found in the FE of excreta and IC varied among the nine experimental groups, being significantly different (*P* < 0.05) for both biological materials of *R. pictipes*. For *R. robustus*, the mean number of PF ranged from 0.0 for G1 (TcIAM), G2 (TcIMG) and G6 (TcIAM+TcIIPR) to 1969.0 for G4 (TcIVAM) in the excreta, and from 0.0 for G1 and G6 to 6885.0 for G5 (TcIAM+TcIVAM) and 5535.0 for G7 (TcIMG+TcIVAM) in the IC (Fig. 2). In *R. pictipes*, no PF were observed in the excreta of six of the nine groups (G2, G4, G5–G7, G9), but the highest mean number of PF (2160.0) was recorded for G6. Of the three groups in which parasites were present in the IC, the mean number of PF in the IC of G3 (TcIIPR) (19,845.0) was significantly higher than that observed in both G8 (TcIMG+TcIIPR; 810.0 PF) and G9 (TcIIPR+TcIVAM; 675.0 PF) (Fig. 2).

The mean number of PF in the IC varied significantly (*P* = 0.0153) between the two triatomine species, with *R. pictipes* groups presenting the highest values, indicating that this species is more susceptible to *T. cruzi* infection than *R. robustus*, especially to the TcI strain from Amazonas, both alone and when associated with the TcII strain. The higher susceptibility to infection of *R. pictipes* in relation to *R. robustus* was also confirmed by the greater mean PF numbers in the TcII infections (Fig. 2).

**Fig. 3:**
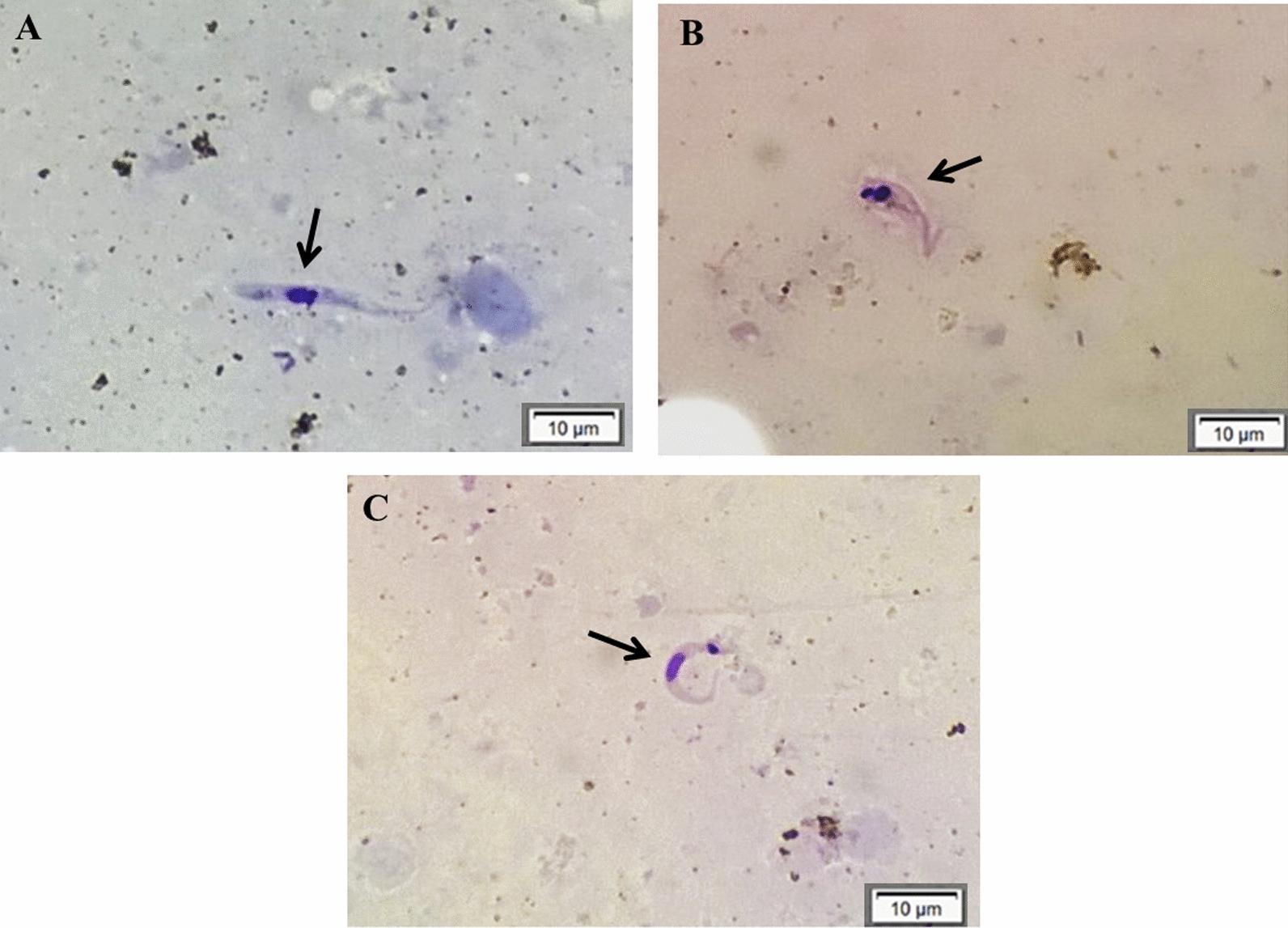
Developmental stages of *T. cruzi* observed by optical microscopy of the intestinal content of an experimentally infected triatomine. **a** Epimastigote, **b** spheromastigote, **c** metacyclic trypomastigote forms. Arrows indicate parasitic forms

After dissection of the insects' digestive tract, it was possible to observe the concomitant presence of MT, EP and SF in the DC (Fig. 3), regardless of the day of infection. The mean number of PF in the DC in the excreta of *R. robustus* ranged from 0.0 for seven of the nine groups (G1-G3, G6-G9) to 13.0 for G4 (TcIVAM) (*P* = 0.005), and in the IC, from 0.0 for G2 (TcIMG) and G6 (TcIAM+TcIIPR) to 40.0 for G4 (*P* = 0.007) (Table 3).

**Table 3:** Mean number of parasitic forms in the differential count of excreta and intestinal contents of *R. robustus* infected with different strains of *T. cruzi*

Experimental group	*Rhodnius robustus*															
	Excreta									IC						
	PF	20 dpi	40 dpi	60 dpi	80 dpi	100 dpi	Total	%MC	*P* value*	30 dpi	60 dpi	90 dpi	120 dpi	Total	%MC	*P *value*
G1 (TcIAM)	EP	0	0	0	0	0	0		–	0	0	0	0	0	–	–
	MT	0	0	0	0	0	0	0.0	–	0	0	0	1	1	100.0	0.003
	SF	0	0	0	0	0	0	–	–	0	0	0	0	0	–	–
G2 (TcIMG)	EP	0	0	0	0	0	0	–	–	0	0	0	0	0	–	–
	MT	0	0	0	0	0	0	0.0	–	0	0	0	0	0	0.0	–
	SF	0	0	0	0	0	0	–	–	0	0	0	0	0	–	–
G3 (TcII)	EP	0	0	0	0	0	0	–	–	1	0	0	3	4	–	–
	MT	0	0	0	0	0	0	0.0	–	1	1	0	2	4	44.4	0.005
	SF	0	0	0	0	0	0	–	–	0	0	0	1	1	–	–
G4 (TcIV)	EP	0	1	0	3	0	4	–	–	16	4	2	1	23	–	–
	MT	4	1	0	2	0	7	53.8	0.005	6	1	1	1	9	22.5	0.007
	SF	1	0	0	1	0	2	–	–		1	0	0	8	–	–
G5 (TcIAM+TcIV)	EP	0	1	1	0	0	2	–	–	0	0	0	1	1	–	–
	MT	0	3	0	0	0	3	50.0	0.004	0	0	1	4	5	62.5	0.007
	SF	0	1	0	0	0	1	–	–	0	0	1	1	2	–	–
G6 (TcIAM+TcII)	EP	0	0	0	0	0	0	–	–	0	0	0	0	0	–	–
	MT	0	0	0	0	0	0	0.0	–	0	0	0	0	0	0.0	–
	SF	0	0	0	0	0	0	–	–	0	0	0	0	0	–	–
G7 (TcIMG+TcIV)	EP	0	0	0	0	0	0	–	–	0	0	4	1	5	–	–
	MT	0	0	0	0	0	0	0.0	–	0	1	3	0	4	21.1	0.006
	SF	0	0	0	0	0	0	–	–	0	1	0	0	1	–	–
G8 (TcIMG+TcII)	EP	0	0	0	0	0	0	–	–	1	0	12	1	14	–	–
	MT	0	0	0	0	0	0	0.0	–	0	0	3	1	4	21.1	0.006
	SF	0	0	0	0	0	0	–	–	0	0	0	1	1	–	–
G9 (TcII+TcIV)	EP	0	0	0	0	0	0	–	–	0	0	0	1	1	–	–
	MT	0	0	0	0	0	0	0.0	–	0	0	0	1	1	0.0	–
	SF	0	0	0	0	0	0	–	–	0	0	0	0	0	–	–
	EP	0	0	0	0	0	0	–	–	0	0	0	0	0	**–**	–

*dpi* Days post-infection, *EP* epimastigote,* %MC* metacyclogenesis rate,* MT* metacyclic trypomastigote,* PF* parasitic forms,* SF* spheromastigote,* –* not applicable

*Shapiro–Wilk test, considering a significance level of 5%

The mean number of PF in the DC for *R. pictipes* excreta ranged from 0.0 for six of the nine groups (G2–G4, G7–G9) to 6.0 for G1 (TcIAM) (*P* = 0.007), and from 1.0 for G5 (TcIAM+TcIVAM) to 189.0 for G4 in the IC (Table 4). For the experimental groups of *R. robustus*, in the first analysis of the excreta performed at 20 dpi, the SF and MT forms for G4 (TcIVAM) were visualized, and these were also visualized at 40 and 80 dpi (Table 3). In the case of mixed infections, MT was observed in the G5 group (TcIAM+TcIVAM) at 40 dpi (Table 3). In *R. pictipes*, the forms EP for G4 and MT for G5 were observed at 20 dpi, while at 60 dpi and 100 dpi, MT and SF were visualized for groups G1 and G6, respectively (Table 4).

**Table 4:** Mean number of parasitic forms in the differential count of excreta and intestinal contents of *R. pictipes* infected with different strains of *T. cruzi*

Experimental group	*Rhodnius pictipes*															
	Excreta									IC						
	PF	20 dpi	40 dpi	60 dpi	80 dpi	100 dpi	Total	%MC	*P* value*	30 dpi	60 dpi	90 dpi	120 dpi	Total	%MC	*P* value*
G1 (TcIAM)	EP	0	0	0	0	2	2	–	–	1	0	30	8	39	–	–
	MT	0	0	1	0	2	3	50.0	0.005	1	0	1	5	7	14.0	0.006
	SF	0	0	0	0	1	1	–	–	2	0	0	2	4	–	–
G2 (TcIMG)	EP	0	0	0	0	0	0	–	–	20	0	6	0	26	–	–
	MT	0	0	0	0	0	0	0.0	–	0	0	1	0	1	3.3	0.003
	SF	0	0	0	0	0	0	–	–	3	0	0	0	3	–	–
G3 (TcII)	EP	0	0	0	0	0	0	–	–	33	22	6	8	69	–	–
	MT	0	0	0	0	0	0	0.0	–	1	3	2	1	7	5.0	0.008
	SF	0	0	0	0	0	0	–	–	5	52	4	2	63	–	–
G4 (TcIV)	EP	0	0	0	0	0	0	–	–	15	144	0	3	162	–	–
	MT	0	0	0	0	0	0	0.0	–	1	2	0	1	4	2.1	0.006
	SF	0	0	0	0	0	0	–	–	3	1	0	19	23	–	–
G5 (TcIAM+TcIV)	EP	1	0	0	0	0	1	–	–	0	0	0	0	0	–	–
	MT	1	0	0	0	0	1	50.0	0.003	1	0	0	0	1	100.0	0.003
	SF	0	0	0	0	0	0	–	–	0	0	0	0	0	–	–
G6 (TcIAM+TcII)	EP	0	0	0	0	0	0	–	–	0	10	9	2	21	–	–
	MT	0	0	0	1	0	1	50.0	0.003	0	1	0	1	2	6.3	0.006
	SF	0	0	0	1	0	1	–	–	0	4	4	1	9	–	–
G7 (TcIMG+TcIV)	EP	0	0	0	0	0	0	–	–	0	0	0	6	6	–	–
	MT	0	0	0	0	0	0	0.0	–	0	0	0	0	0	0.0	–
	SF	0	0	0	0	0	0	–	–	0	0	0	0	0	–	–
G8 (TcIMG+TcII)	EP	0	0	0	0	0	0	–	–	1	0	6	0	7	–	–
	MT	0	0	0	0	0	0	**–**	–	1	0	0	0	1	12.5	0.003
	SF	0	0	0	0	0	0	**–**	–	0	0	0	0	0	**–**	–
G9 (TcII+TcIV)	EP	0	0	0	0	0	0	**–**	–	0	0	0	0	0	**–**	–
	MT	0	0	0	0	0	0	0.0	–	0	1	1	0	2	100.0	0.004
	SF	0	0	0	0	0	0	**–**	–	0	0	0	0	0	**–**	–

*Shapiro–Wilk test, considering a significance level of 5%

### Metacyclogenesis rates

Metacyclogenesis rates (%MC) in excreta for G4 (TcIVAM) and G5 (TcIAM + TcIVAM) of *R. robustus* were significantly higher, 53.8% (*P* = 0.005) and 50.0% (*P* = 0.004) respectively, than those in the other groups for this vector species. This demonstrates vector competence of *R. robustus* to TcIV, in both pure infection and mixed infection with TcIAM (Table 3). In the IC of this species, MT forms were found in both the pure infection groups (G1, G3, and G4) and mixed infection groups (G5, G7, G8, and G9) over the course of infection (Table 3).

MT forms were already observed at 30 dpi for G3 (TcIIPR) and G4, and the number of MT ranged from 0.0 for G2 (TcIMG) and G6 (TcIAM+TcIIPR) to 40.0 for G4. *Rhodnius robustus* was susceptible to pure infection with both TcII and TcIV as determined by the IC examination, and the highest percentages of MT forms recorded in this biological material were, in descending order, G5 (TcIAM+TcIVAM; 62.5%, *P* = 0.007) > G3 (TcIIPR; 44.4%, *P* = 0.005) > G4 (TcIVAM; 22.5%, *P* = 0.007) > G7 (TcIMG+TcIVAM; 21.1%, *P* = 0.006) > G8 (TcIMG+TcIIPR; 21.1%, *P* = 0.006) (Table 3).

Regarding *R. pictipes*, MT forms were observed in the excreta at 20 dpi for G5 (TcIAM+TcIVAM) (Table 4), indicating vector competence of this species for the Amazonas strains in mixed infection. MT forms were also observed in the excreta from G1 (TcIAM) at 60 and 100 dpi and from G6 (TcIAM+TcIIPR) at 80 dpi (Table 4). The %MC observed for these three groups (G1, G5 and G6) was 50.0%, which is significantly higher than the %MC in the other groups (*P* ≤ 0.005), demonstrating vector competence of this species for TcI from Amazonas both in pure and mixed infection with TcIV and TcII (Table 4). The highest percentages of MT forms (100.0%) were recorded for G5 (*P* = 0.003) and G9 (TcIIPR+TcIVAM) (*P* = 0.004) (Table 4). However, these two groups presented only one and two MT forms in the excreta, respectively.

The %MC in the groups of *R. pictipes* with a considerable number of PF in the excreta were, in descending order, 14.0% in G1 (TcIAM) > 12.5% in G8 (TcIMG+TcIIPR) > 6.3% in G6 (TcIAM+TcIIPR) > 5.0% in G3 (TcIIPR), indicating the vector competence of the species for TcI from Amazonas and TcII, both in pure and mixed infections (Table 4). In the *R. pictipes* IC, all three parasitic forms (EP, MT and SF) were observed for the four pure infection groups and only for G6 among the mixed infection groups (Table 4).

### *Trypanosoma cruzi* genotyping

A RFLP-PCR of the COXII gene did not amplify *T. cruzi* DNA in any of the IC samples tested, despite numerous attempts, including restriction enzyme replacement. Thus, it was not possible to genotype the DTUs with this molecular marker for this type of biological material.

**Fig. 4:**
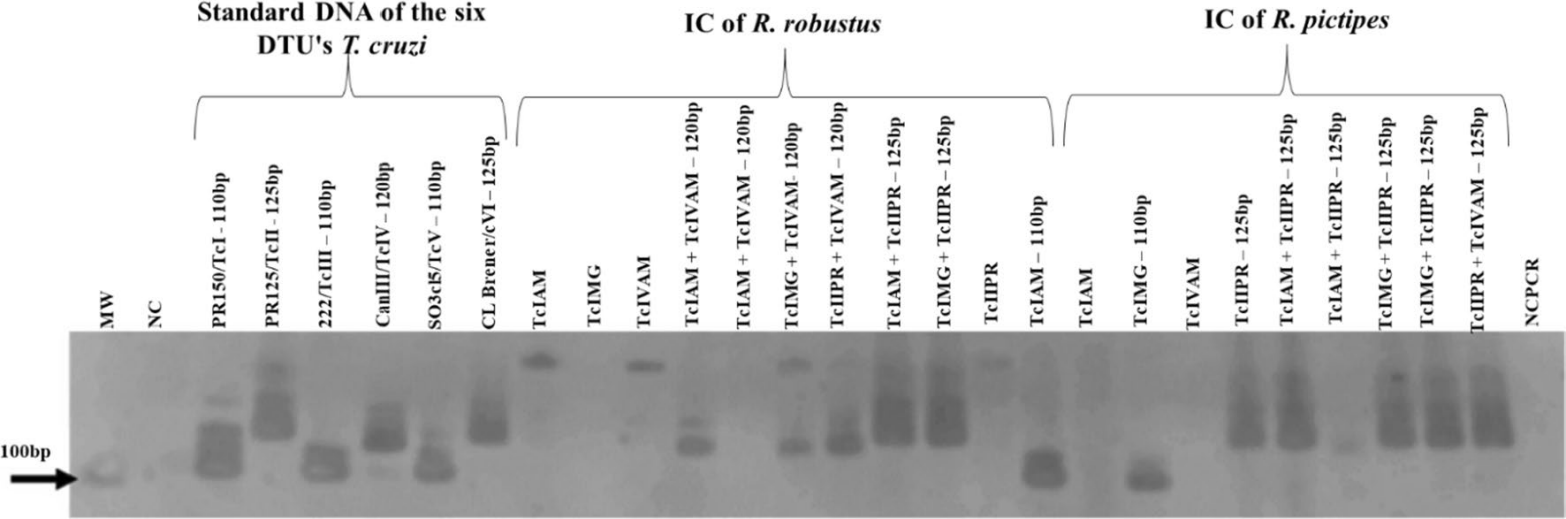
Silver staining of products in 6% polyacrylamide gel electrophoresis show the 24Sα rDNA gene of *T. cruzi* strains (TcIAM, TcIMG, TcIIPR and TcIVAM) in the intestinal contents of *R.* *robustus* and *R. pictipes* with pure and mixed infections. Lanes (from left to right): 1, 100-bp molecular weight (MW) standard; 2, negative control (NC); 3–8, standard DNA of the 6 *T. cruzi* DTUs (TcI–TcVI); 9–11, 18, 20 and 22, negative samples for *T. cruzi*; 12, TcIAM+TcIVAM (G5); 14, TcIMG+TcIVAM (G7); 15, TcIIPR+TcIVAM (G9); 16, TcIAM+TcIIPR (G6); 17, TcIMG+TcIIPR (G8); 19, TcIAM (G1); 21, TcIMG (G2); 23, TcIIPR (G3); 24, 25, TcIAM+TcIIPR (G6); 26, 27, TcIMG+TcIIPR (G8); 28, TcIIPR+TcIVAM (G9); 29, negative control for PCR (NCPCR; no DNA). Abbreviations: DTUs, discrete typing units

On the other hand, PCR genotyping of the *T. cruzi* 24Sα rDNA gene could be performed only for the IC samples (Fig. 4). In pure infections, the 110-bp band could be detected for TcIAM in the *R. robustus* IC (lane 19) and for TcIMG (lane 21) and the 125-bp band for TcIIPR (Lanes 23) in the *R. pictipes* IC (Fig. 4). In the mixed infection groups (G5-G9) of both *Rhodnius* species, DNA fragments of 120 or 125 bp were detected, characteristic of TcII and TcIV, respectively (Fig. 4).

### Infectiousness and mortality

The results of the four techniques used demonstrated that the infectivity rates (%INF) of the four *T. cruzi* strains for *R. robustus* and *R. pictipes*, in pure and mixed infections, were 100.0% for all experimental groups, regardless of the biological material used in the analyses.

Mortality rates (%MOR) for the experimental groups with the two triatomine species ranged from 5.0% to 30.0%, with no significant difference. In pure infections, the highest %MOR recorded were for G1 and G2 (25.0% and 30.0%, respectively) of *R. pictipes*, and for *R. robustus* it was 20.0% for G1. The highest %MOR in mixed infections were 25.0% for G6 and G7 in *R. pictipes* and 20.0% for G8 in *R. robustus*, regardless of DTU and geographic origin (Table 5).

**Table 5:** Mean mortality rate for the nymphs of *R. robustus* and *R. pictipes* with pure and mixed infections of* Trypanosoma cruzi* I, II and IV strains

Experimental groups	*Rhodnius robustus* (%)	*Rhodnius pictipes* (%)
G1 (TcIAM)	20.0	25.0
G2 (TcIMG)	15.0	30.0
G3 (TcIIPR)	10.0	20.0
G4 (TcIVAM)	15.0	5.0
G5 (TcIAM+TcIVAM)	10.0	15.0
G6 (TcIAM+TcIIPR)	5.0	25.0
G7 (TcIMG+TcIVAM)	5.0	25.0
G8 (TcIMG+TcIIPR)	20.0	10.0
G9 (TcIIPR+TcIVAM)	5.0	15.0

Values presented in table are mean mortality rates

## Discussion

In the present study, the influence of the genetic diversity (DTU) of *T. cruzi* on the susceptibility to infection and vector competence of two different species of triatomines belonging to the genus *Rhodnius* was evaluated. Under controlled conditions, it was possible to observe *T. cruzi* infection by different strains in fifth-instar nymphs of *R. robustus* and *R. pictipes*. Furthermore, both *Rhodnius* species were susceptible (100.0% infectivity) to *T. cruzi* strains belonging to the TcI, TcII, and TcIV DTUs.

The amount of blood and BT ingested varied between the two triatomine species. Considering the groups of insects that ingested the highest amount of BT forms, the fifth-instar nymphs of *R. robustus* from G1 (TcIAM) ingested, on average, 76,720 BT, while those of *R. pictipes* from G3 (TcIIPR) ingested a mean of 48,960 BT. This difference in the volume of blood ingested and, consequently, in the amount of infective forms, is related to morphometric differences between the nymphs of the two species. Adult insects of the *R. robustus* species are known to be larger, measuring between 20 and 26 mm in length, while the adult insects of *R. pictipes* are between 18 and 22 mm in length [47]. Barreto-Santana et al. [48] compared the blood intake between the different developmental stages of *R. robustus* and *R. neglectus* and reported that the highest intake was for the fifth-instar nymphs of *R. robustus*, while Rubio et al. [49] showed that fifth-instar nymphs of *R. robustus* ingest, on average, 337.19 mg of blood. However, for *R. pictipes* in the same nymphal stage, it has been reported that blood intake ranges from 127.40 to 199.50 mg [20, 32]. Therefore, the data in the present study show that *R. robustus* can ingest up to twice the volume of blood ingested by *R. pictipes* (730.0 vs. 465.0 mg), corroborating the results of other authors [20, 32, 48, 49].

The susceptibility to infection of *R. robustus* evaluated by the FE in the IC was higher for the TcIVAM strain, since the highest mean number of PF (1890.0) recorded was in the pure infection. Although the TcIAM strain originates from the same geographic area as *R. robustus*, PF were not observed in the IC of the nymphs infected by this strain. These data demonstrate that *R. robustus* is susceptible to infection by TcIVAM, but not to infection by TcIAM, suggesting that the genetic constitution of *T. cruzi* also influences this parameter. The mixed infection of both these Amazonas strains (G5) presented the highest number of PF in the IC (6885.0), suggesting a synergism between the two strains. Synergism seems to have also occurred between TcIVAM and TcIMG, which, when mixed (G7), had the second highest number of PF in the IC (5530.0). There was a predominance of TcIVAM when associated with TcIAM, TcIMG and TcIIPR, as observed in the rDNA-PCR genotyping that detected this DTU in the IC of *R. robustus*. These data show that the genetic lineage of the parasite influences the susceptibility to infection of the vector in the case of mixed infection. In the Brazilian Amazon, both TcI and TcIV have been isolated from naturally infected *R. robustus* and found to be associated with oral CD cases [28, 42, 50].

*Rhodnius pictipes* was observed to be susceptible to pure infection with the four *T. cruzi* strains studied (AM33, PR150, PR1256, and AM14), presenting the highest values in the mean number of PF in the IC of the entire study. The highest susceptibilities occurred both for the TcIAM (22,680.0 PF) and TcIVAM (10,665.0 PF) strains, from the Amazon region, and for the Paraná TcIIPR (19,845.0 PF) and Minas Gerais TcIMG strains (5670.0 PF). These data suggest that the susceptibility of *R. pictipes* to *T. cruzi* is also influenced by the genetics of the parasite, since this triatomine species, like *R. robustus,* was susceptible to the TcI strain from Minas Gerais. The number of PF observed in the IC of *R. pictipes* following infection with the mixed TcIAM and TcIIPR inoculum (17,145.0 PF) shows susceptibility but suggests a negative interaction between these two DTUs since the number of PF in the mixed infection was lower than the simple sum of the PF number for the pure infections. The lowest number of PF recorded for the other groups of *R. pictipes* in mixed infections with TcIMG+TcIVAM (2960.0 PF), TcIAM+TcIIPR (810.0 PF) and TcIIPR+TcIVAM (675.0 PF) also indicates antagonistic interactions*.* These values were lower−or even null (TcIAM+TcIVAM) in the case of G5−than those presented by the same strains alone in the pure infection groups (TcIAM, TcIMG, TcIIPR, and TcIVAM), suggesting partial or total inhibition of the parasite population in mixed infections. The genotyping data also showed that in mixed infections of both vector species in which TcI was present, this DTU was inhibited or eliminated by the other DTU and thus was not detected. A review of the literature showed that in nature, TcI has been the only DTU isolated from *R. pictipes*, as well as from the species *Rhodnius pallescens*, native to Colombia [7, 28, 51].

The vector competence, evaluated both by the presence and number of PF and by the %MC, in the excreta varied between the vector species and the *T. cruzi* strain. Based on the parameter “number of PF in the excreta” of *R. robustus* with pure infection, it was clear that the interactions between *R. robustus* and TcIVAM was greater (1969.0 PF) than that with all other groups, but no PF were observed in the *R. robustus* excreta for the TcIAM and TcIMG groups. These data demonstrate a greater influence of the parasite's genetics on the vector competence of this triatomine species; that is, in pure infections, *R. robustus* is able to eliminate greater amounts of PF from the TcIV strain in the excreta, but not from the TcI strains. The lowest values of the PF number in the excreta were recorded for TcI, regardless of geographic origin, in pure and mixed infections, except for the TcIAM+TcIVAM group (G5). Among the mixed infections in this vector, the infection with the TcIAM+TcIVAM strains had the highest number of PF in the excreta (560.0). However, this number was lower than the sum of the PF number of the pure infections with the same strains, suggesting that, although there is a negative interaction between the two Amazonas strains in *R. robustus*, leading to a reduction in the number of PF in the excreta, this species continues to eliminate PF along with the excreta, and may present vector competence for TcIAM+TcIVAM.

For *R. pictipes*, the results of the parameter “number of PF in the excreta,” indicate that PF elimination occurred only with TcI from Amazonas and TcII from Paraná, in both pure and mixed infections with these two strains. The highest PF values were observed in the excreta of this species, although the number of PF was lower than that observed in the IC; that is, for the TcI DTU, *R. picitipes* showed vector competence for the Amazonas strain, but not for the Minas Gerais strain, in addition to vector competence for TcII, whose geographic origin (Paraná) is different from the geographic area of occurrence of this vector species (*R. pictipes*, Amazon region). One implication of these experimental data is that this triatomine species could transmit TcII strains from another region when introduced in the Brazilian Amazon region.

Another important parameter to determine vector competence that also has an impact on the transmission of *T. cruzi* is the %MC. This metric determines the quality of the vector and also influences the transmission dynamics of the parasite; that is, the higher the %MC, the more effective the transmission of *T. cruzi* to the vertebrate host [2]. The %MC must be determined both in the excreta and in the IC, because if infected insects are present during food processing, infective parasite forms in the excreta or the IC could contaminate the product when the insect is crushed. The results obtained for this parameter also suggest the influence of geographic origin on vector competence, since the highest %MC values were found in the excreta of *R. robustus* with TcIVAM (53.8%) and *R. pictipes* with TcIAM (50.0%) in pure infections, and in the IC of both *R. pictipes* (100%, only 1 MT form was observed) and *R. robustus* (62.5%) for the mixed infections of TcIAM+TcIVAM. An exception was the *R. pictipes*/TcIIPR+TcIVAM group which also had a %MC of 100% (only 2 MT forms were observed), even in the mixture a *T. cruzi* strain with a different geographic origin from the area where the vector species occurs. In the IC, the highest %MC (62.5%) was also recorded for *R. robustus*/TcIAM+TcIVAM. These data also suggest that these vector species showed high efficiency in transforming proliferative forms (EP) of *T. cruzi* into infective forms (MT). High efficiency in metacyclogenesis has also been observed in domestic species of *Triatoma infestans*, *R. prolixus*, and *Panstrongylus megistus*, where the %MC ranged from 60.0% to 70.0% for different DTU strains [2].

The interaction between the TcII strain from Paraná and the *R. pictipes* vector species in this study deserves mention. Even though this DTU is not frequently isolated in the Amazon region, the presence of this vector species has been reported beyond the borders of Brazil's Legal Amazon (states of Acre, Amazonas, Maranhão, Mato Grosso, Pará and Tocantins), such as the states of Piauí, Mato Grosso do Sul and Minas Gerais [32, 52]. The wide geographic distribution of this vector species increases the possibility of it encountering TcII strains in the Central-West, Southeast and South macro-regions of Brazil.

Our results show that strains of different DTUs tend to behave differently in both *R. robustus* and *R. pictipes*; that is, the interactions between the DTUs studied in these vectors differ in their ability to complete their biological cycle in the digestive tract. The best interactions occurred between *T. cruzi* strains and triatomine species of the same geographic origin, likely because the vector and the parasite are better adapted to each other due to a long period of co-evolution [31]. Consequently, these parasites are able to become established and replicate efficiently in the vector and still produce infective forms, which is important in maintaining the life-cycle of the parasite in nature [26, 53, 54].

The genotyping of insect IC by rDNA-PCR showed that TcII and TcIV DTUs predominated (or overlapped TcI DTU) in the associations for both *Rhodnius* species. These results may be due the infective meal being performed on mice, where the host immune system, especially the cells of the mononuclear phagocytic system, could select parasite populations of one DTU to the detriment of another [55, 56]. That is, the parasite populations in the circulating blood of the mouse may be at different proportions and, consequently, the insect could feed and become infected with different proportions of each of the two strains [56]. This selection of the *T. cruzi* population that occurs may also be related to intrinsic factors of triatomines (components present in saliva, lysozymes, defensins, etc.) that can inhibit the development of a certain genetic lineage of the parasite, as occurs with the Y (TcII) lineage in *R. prolixus*, where the components present in the insect's saliva inhibit the development of this strain [27, 57–60].

The parasitological techniques (FE and DC) used in this study, as well as *k*DNA-PCR, proved to be efficient for analyzing both biological materials. Among the techniques used, *k*DNA-PCR presented the highest sensitivity, being able to detect the DNA of the parasite both in the excreta and in the IC samples at greater proportions of the insects: from 50.0% (IC) to 100.0% (excreta) for *R. robustus*, and from 37.5% (IC) to 100.0% (excreta) for *R. pictipes*. This difference in *k*DNA-PCR sensitivity, observed between the two biological materials analyzed, may be associated with a greater presence of PCR inhibitors in the IC of these insects.

Genotyping by analysis of RFLP of the COXII gene, which involves cutting DNA fragments with the enzyme AluI, was also tested in the present study. However, this analysis showed partial or unspecific cleavage in the IC samples (data not shown). Our results differ from those reported by Sá et al. [44] for *R. prolixus* and suggest that both *Rhodnius* species studied may have components that inhibit the AluI activity. On the other hand, PCR of the 24Sα rDNA gene was able to detect and characterize genetic material only in the IC samples. This difference in detection capacity between *k*DNA-PCR and rDNA–PCR may be related to the amount of parasitic forms present in the samples and to the number of copies of the gene of interest. The* k*DNA minicircle is represented in thousands of copies, with an approximate size of 1.4 kb, and is considered to be a naturally amplified DNA [61–63].

## Conclusions

This study showed that the two species of *Rhodnius* analyzed have vector capacity (susceptibility to infection and vector competence). *Rhodnius robustus* showed vector competence for pure infection of TcIVAM and mixed infection of TcIAM+TcIVAM, and *R. pictipes* showed vector competence for TcIAM+TcIVAM and TcIAM+TcIIPR. Early elimination of infective forms was observed for *R. robustus*, which increases its efficiency as a vector. Taken together, the data suggest that the genetics of the parasite and its geographic origin influence the susceptibility to infection and vector competence in pure and mixed infections of two triatomine species from the Amazon region.

## Data Availability

The datasets generated and/or analyzed during the current study are not publicly available (all data are shown in our results), but they are available from the corresponding author upon reasonable request.

## Abbreviations

BT: Blood trypomastigote
CD: Chagas disease
CMT: Culture-derived metacyclic trypomastigotes
dpi: Days post-infection
DC: Differential count
DTUs: Discrete typing units
EP: Epimastigote
FE: Fresh examination
IC: Intestinal content
%INF: Infection rate
*k*DNA: Kinetoplast DNA
LIT: Liver infusion tryptose
%MC: Metacyclogenesis rate
%MOR: Mortality rate
MT: Metacyclic trypomastigote
PF: Parasitic forms
PBS: Phosphate-buffered saline
SF: Spheromastigote
rDNA: ribosomal DNA
